# Potential protective effect of astaxanthin on ovary ischemia-reperfusion injury

**DOI:** 10.22038/IJBMS.2022.61289.13559

**Published:** 2022-02

**Authors:** Erdem Toktay, Tugba Bal Tastan, Muhammet Ali Gurbuz, Elif Erbas, Ozlem Demir, Rustem Anıl Ugan, Jale Selli

**Affiliations:** 1 Kafkas University, Faculty of Medicine, Department of Histology and Embryology, Kars, Turkey; 2 Ataturk University, Faculty of Medicine, Department of Histology and Embryology, Erzurum, Turkey; 3 Ataturk University, Faculty of Pharmacy, Department of Pharmacology, Erzurum, Turkey; 4 Alanya Alaaddin Keykubat University, Faculty of Medicine, Department of Histology and Embryology, Antalya, Turkey

**Keywords:** Astaxanthin, Ischemia, Ovary, Oxidative stress, Rat, Reperfusion

## Abstract

**Objective(s):**

We thought that astaxanthin (ASX) might be a protective agent in oxidative stress damage that develops against ischemia and reperfusion injury in the rat ovary.

**Materials and Methods:**

The experimental groups consisted of healthy, I (Ischemia), I+ASX50, I+ASX100, I/R (Ischemia/Reperfusion), I/R+ ASX50, and I/R+ ASX100. Vascular clamps were applied to the ovaries for 3 hr to induce ischemia. For the reperfusion groups, the clamps were opened and blood flow was restored to the ovaries for 3 hr. At the end of the experiment, biochemical, histopathological, and immunohistochemical analyses were made from the tissue samples taken.

**Results:**

While MDA levels increased significantly in I and I/R groups, SOD levels decreased. It was found that ASX significantly decreased MDA levels and increased SOD activity in treatment groups depending on the dose. Caspase 3, IL-1 β, and IL-6 expressions were severely increased in ischemia and I/R groups, while the severity of I+ASX50 and I/R+ASX100 immunoreactivity was decreased. While severe hemorrhage areas were observed in I and IR groups, minimal hemorrhage areas were observed in the treatment groups, especially in I/R+ASX100 groups. In addition, inflammatory cells and necrotic cells in the I/R group were not observed in I/R+ASX50 and I/R+ASX100 groups.

**Conclusion:**

As a result, it was determined that ASX has a strong protective role against oxidative damage in the treatment of ovarian ischemia-reperfusion injury.

## Introduction

Ovarian torsion decreases or causes complete cessation of blood flow due to rotation of ovary around the suspensor ligament. It can be caused by previous surgeries in the pelvic-abdominal area, pregnancy, trauma, and ovarian cysts, and is in the 5th place (2.7–7.4%) among all gynecological emergencies (1, 2). Early diagnosis is critical in treatment. If the blood flow is not urgently provided again, hemorrhage and edema occur in the ovary. This problem requires surgical operation and detorsion (3). However, re-flow of blood to the ovarian tissue leads to the development of reperfusion damage and exacerbation of oxidative damage (4). Reactive oxygen molecules (ROS) particularly damage nucleic acid (DNA and RNA), structural protein, and cell membranes (4). The exacerbated oxidative damage impairs ovarian tissue and organ function. It can lead to temporary or permanent infertility (5). At this point, the anti-oxidant defense system plays an important role in the cell. However, these cellular anti-oxidant defense systems are insufficient in cases of ovarian ischemia and reperfusion injury (6). Therefore, high anti-oxidants such as beta-carotene, folic acid, and melatonin, which are supplemental food, are necessary to prevent organ damage.

Astaxanthin (ASX), a well-known strong anti-oxidant, is a xanthophyll-derived carotenoid pigment. It is abundant in single-celled yeast and microalgae, in fish such as salmon, trout, and red perch, in shelled invertebrates such as krill, and crayfish shrimp containing red pigment (7, 8). Studies have shown that the capacity of ASX to clean reactive oxygen derivatives is 550 times more effective than Vitamin E, 800 times more effective than coenzyme Q10, and 6000 times more effective than Vitamin C (9). In addition, ASX has a lipophilic structure and the transmembrane structure stands out from other anti-oxidants (10). Previous studies demonstrated that ASX prevents the kidney (11), retina (12), and liver (13) against ischemia and reperfusion damage via exerting an anti-oxidant effect. However, there is no study about the effect of ASX on ovarian ischemia and reperfusion injury.

Briefly, in our study, we investigated that ASX might be a protective agent against ischemia and reperfusion injury in the rat ovary.

## Materials and Methods

***Ethics statement and animals***

 The Institutional Animal Care and Use Ethics Committee of Kafkas University approved the study on 26.12.2019, which was conducted in accordance with protocol number 2019/153. Forty-two Sprague-Dawley female rats, with average weight of 200–250 grams and 10–12 weeks of age were obtained from Ataturk University Medicinal and Experimental Application and Research Center, Erzurum, Turkey (ATADEM). The rats were given enough (*ad libitum*) water and pellet feed during the experiment. They were housed at optimal room temperature and humidity level.

***Surgical procedures and animal experimental model***

The animals were randomly divided into 7 groups (n=6). The experimental groups consisted of Healthy, I (Ischemia), I+ASX50, I+ASX100, I/R (Ischemia and Reperfusion), I/R+ ASX50, and I/R+ ASX100. On the day of the experiment, the animals were anesthetized with a mix of ketamine (Ketalar, Phizer) and xylazine (Rometar, Bioveta). An incision was made in the abdominal area of each animal to view the ovaries. Vascular clamps were applied to the ovaries for 3 hr to induce ischemia. After 3 hr, the ischemia groups were terminated. For the reperfusion groups, the clamps were opened and blood flow was restored to the ovaries for 3 hr. Reperfusion groups were terminated after 3 hr. Healthy group rats underwent laparotomies without the induction of I and I/R injuries. To investigate the protective effect of ASX (Acros Organics, 1 g, lot no: A0402491) on ovarian tissue with induced I/R injury, ASX was orally (dissolved in distilled water) given at doses of 50 and 100 mg/kg during both I and I/R (1 hr prior to surgery) (14, 15). 

At the end of the experiment, all animals were anesthetized through IP administration of a combination of 15 mg/kg xylazine and 100 mg/kg ketamine. A longitudinal incision (3 cm) was created in the midline area of the lower abdomen. The structure of uterine horns was observed and ovary organs were carefully kept via pens. Collected tissues were stored at 3.7 % formaldehyde for histopathological and immunohistochemical analyses. In addition, tissue samples were stored at -80 °C for biochemical examination.

***Histolologic analyses***

Ovarian Tissues were rapidly fixed in 3,7 % formaldehyde solution for 48 hr. After fixation, all ovary samples for histological tissue processing were routinely performed as previously described (16). After tissue processing, 5 micrometer thick sections were taken from each paraffin block for histopathological and immunohistochemical examination. Ovarian tissue slides were stained with Hematoxylin-Eosin.

Immunohistochemical staining was performed using the Ventana BENCHMARK GX automatic immunohistochemistry staining system (17). We used monoclonal primary antibodies (IL 1ß, IL 6, and Caspase3, Santa Cruz) in a 1:100 dilution. All slides were photographed by a Nikon Eclipse e600 microscope with a computer-aided camera.

***Biochemical analyses***

All tissue samples from each rat were ground in liquid nitrogen using a TissueLyser II device (Qiagen, Hilden, Germany). Then, 100 mg tissue was taken from each tissue and homogenized in 1 ml phosphate buffer solution. Subsequently, The samples were centrifuged and for SOD activity, and MDA level, each supernatant was measured with highly sensitive kits (BT LAB - E0168Ra, E0156Ra (China), respectively, specifically designed for rat tissue, according to the manufacturer’s instructions. The protein concentrations were determined by the Lowry method using commercial protein standards (Sigma Aldrich, Total protein kit-TP0300-1KT, USA). All data were presented as the mean ± standard deviation (SD) based on per mg of protein (14).

***Statistical and semi-quantitative analysis***

The data of our study were statistically evaluated with IBM 20.00 SPSS program. The groups were compared with Tukey post-hoc tests and one-way ANOVA multiple comparison test.

In immunohistochemical staining, semi-quantitative scoring was performed. At least five areas were evaluated for each ovary slide and the average staining density score was taken into account (18, 19).

## Results

***Histopathological findings***

*Hematoxylin and eosin staining findings*

In the healthy group, the cortex and medulla of the ovary were examined. Healthy primary, secondary, and graf follicles and corpus luteum structures in different sizes and periods were observed in the cortex (Figure 1 A–D).

In the ischemia group, intense hemorrhage areas were observed in the cortex. The areas of edema were remarkable. Furthermore, apoptotic cells were found in granulosa cells in the follicle (Figure 2 A–B). In the I+ASX50 group, Intense hemorrhage areas were observed in the cortex, and edema and apoptotic cells were not found (Figure 2 C–D). In the I+ASX100 group, dense and minimal hemorrhage areas were observed around the follicle in the cortex. In this group, hemorrhage areas decreased significantly compared with the ischemia group. Edema and apoptotic cells were also not found (Figure 2 E–F).

In the I/R group, intense hemorrhage areas were observed in the cortex. Necrotic cells were found in the follicles. In addition, edema and inflammatory areas were detected (Figure 3 A–B). Minimal hemorrhage areas were seen in the cortex in the I/R+ASX50 group. Edema and apoptotic cells were not seen (Figure 3 C–D). Minimal hemorrhage areas were seen in the cortex in the I/R+ASX 100 group. While apoptotic and necrotic cells were not observed, hemosiderin-laden macrophages were found in areas where hemorrhage was decreased (Figure 3 E–F).

The histopathological scoring was performed considering the hemorrhagic and edematous areas observed in the ovary, presence of necrotic cells, and accumulation of inflammatory cells (Table 1).

*Immunohistochemical findings*

The immunohistochemical results of the study were scored considering the degree of immunity (Table 2).

**Figure 4.**
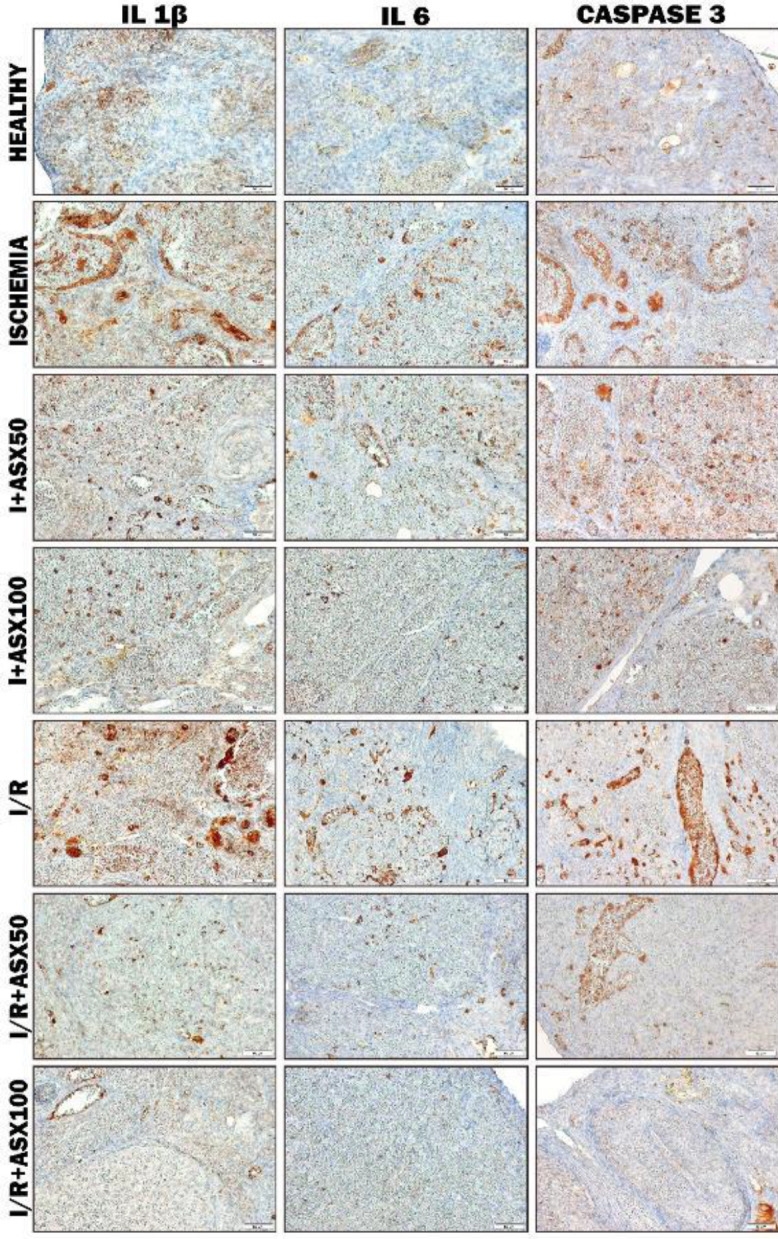
IL 1β, IL 6, and Caspase 3 immunohistochemical findings in rat ovary (I: Ischemia, I/R: Ischemia and reperfusion, ASX50: Astaxanthin 50 mg/kg, ASX100: Astaxanthin 100 mg/kg, IL 1 β: Interleukin 1 beta, IL 6: Interleukin 6)

According to immunohistochemistry staining results in IL 1β antigen staining findings, I and I/R groups were severe immune positive, the I+ASX50 group was moderate immune positive, I+ASX100 and I/R+ASX50 groups were mild immune positive; there was little or no immunopositivity in the HEALTHY group, while the I/R+ASX100 group was immune negative (Figure 4).

In IL 6 antigen staining findings, I and I / R groups were moderate immune positive, I+ASX50 groups were mild immune positive, in the I/R+ASX50 group was little or no immune positivity; HEALTHY, I+ASX100, and I/R+ASX100 groups were immune negative (Figure 4).

In CASPASE 3 antigen staining findings, I and I/R groups were severely immune positive, the I+ASX50 group was moderately immune positive, I+ASX100 and I/R+ASX50 groups were mildly immune positive, while in I/R+ASX100 and HEALTHY groups immune negativity was seen (Figure 4).

*Biochemical findings*

**Figure 5.**
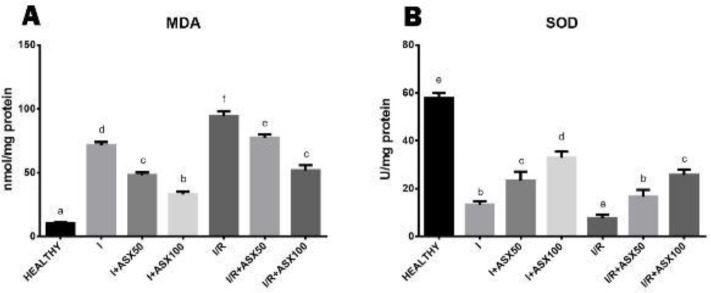
**MDA and SOD levels of all experimental groups in rat ovary tissue** (I: Ischemia, I/R: Ischemia and reperfusion, ASX50: Astaxanthin 50 mg/kg, ASX100: Astaxanthin 100 mg/kg, IL 1 β: Interleukin 1 beta, IL 6: Interleukin 6)

MDA levels in I and I/R groups were higher than in the healthy group. These levels were significantly reduced in a dose-dependent manner in ischemia and I/R+ASX treatment groups. (Figure 5-A). We determined that the SOD level was reduced in the ischemia group. But, it increased in the ischemia treatment groups (I+ASX50 and I+ASX100). SOD levels were especially low in I/R groups. SOD activity increased in a dose-dependent manner in ASX treatment groups (I/R+ASX50 and I/R+ASX100) (Figure 5-B).

**Fıgure 1:**
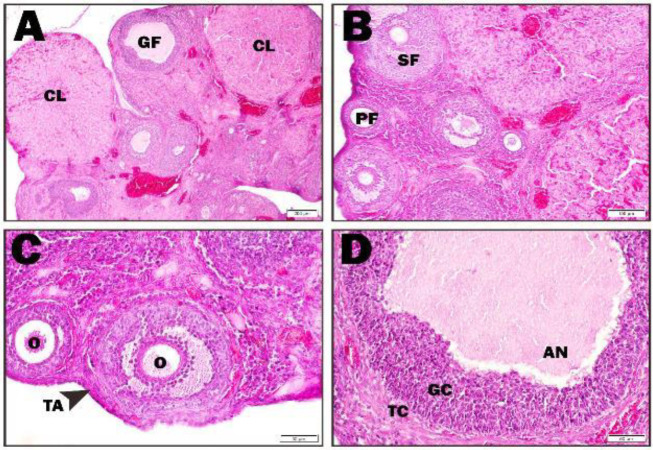
Healthy group histopathological findings in rat ovary (A-D: Healthy groups;CL: Corpus luteum, GF: Grafian follicul, SF: Secondary follicul, PF: Primary follicul, TA: Tunika albugenea, O: oosit, An: Antrum, GC: Granulosa cell, TC: Teka cell)

**Fıgure 2:**
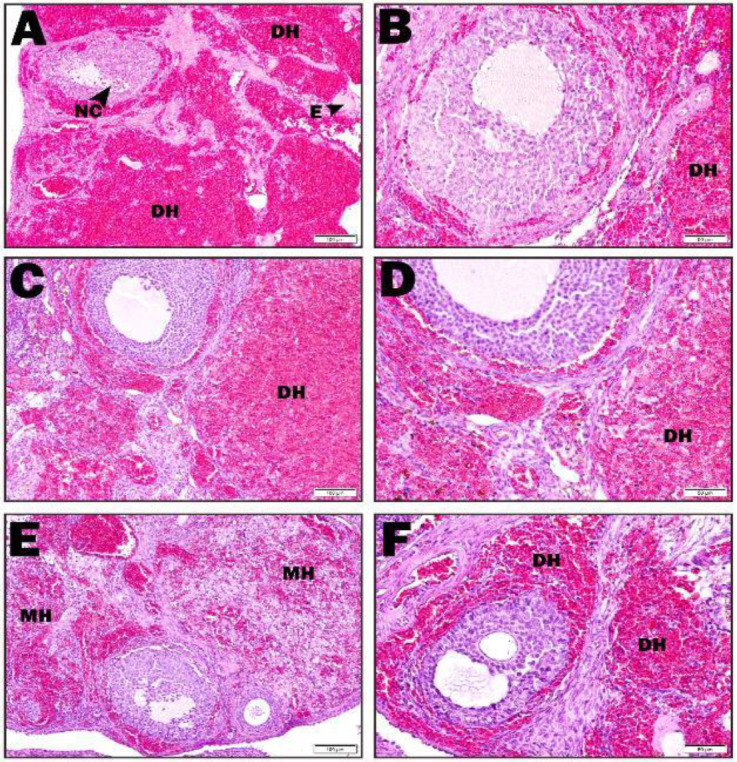
Ischemia, I+ASX50, and I+ASX100 groups histopathological

findings in rat ovary (A-B: ischemia group, C-D: I+ASX50 group, E-F: I+ASX100 group; NC: Necrotic cell, DH: Dense hemorrhage, E: Edema, MH: Minimal hemorrhage)

**Fıgure 3:**
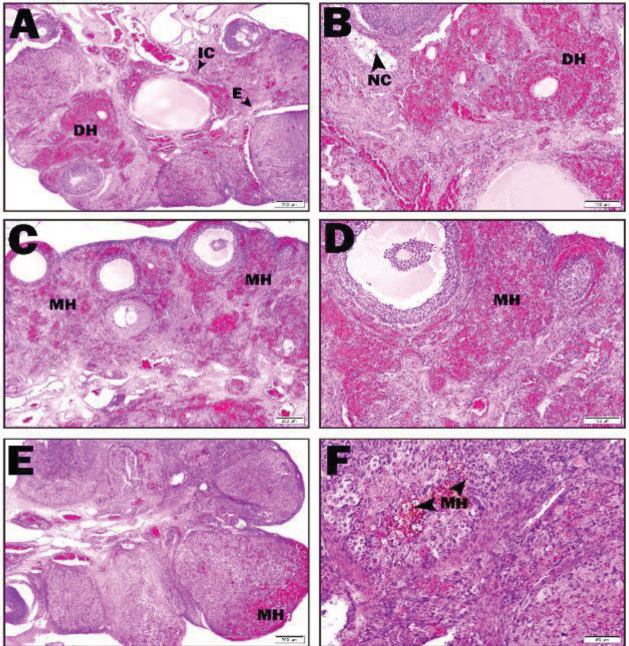
I/R, I/R+ASX50 and I/R+ASX100 groups histopathological

findings in rat ovary (A-B: I/R group, C-D: I/R +ASX50 group, E-F: I/R +ASX100 group; IC: Inflammatory cell, NC: Necrotic cell, DH: Dense hemorrhage, E: Edema, MH: Minimal hemorrhage)

**Table 1:** Histopathological scoring findings of experimental groups ( Healthy, I (Ischemia),I+ASX 50, I+ASX100, I/R (Ischemia and Reperfusion), I/R+ ASX 50, and I/R+ ASX100)

Groups	Hemorrhage	Edema	Necrotic cells	Inflammatory cells
Healthy	-	-	-	-
Ischemia	+++	++	++	-
I+ASX50	++	-	-	-
I+ASX100	+	-	-	-
I/R	+	+	+	+
I/R+ASX50	-/+	-	-	-
I/R+ASX100	-	-	-	-

Note: histopathological scoring was performed considering the hemorrhagic and edematous areas observed in the ovary, presence of necrotic cells, and accumulation of inflammatory cells; symbolized to be absent (-), mild (+), moderate (++), and severe (+++) damage

**Table 2:** IL 1β, IL 6 and Caspase 3 immunohistochemical scoring findings of experimental groups ( Healthy, I (Ischemia),I+ASX 50, I+ASX100, I/R (Ischemia and Reperfusion), I/R+ ASX 50, and I/R+ ASX100)

Groups	IL 1β	IL 6	Caspase 3
Healthy	-/+	-	+
Ischemia	+++	++	+++
I+ASX50	++	+	++
I+ASX100	+	-	+
I/R	+++	++	+++
I/R+ASX50	+	-/+	+
I/R+ASX100	-	-	-

Note: Immune positivity was scored with – (0%) for little or no, + (0–30%) for mild, ++ (30–60%) for moderate, and + + + (60–100%) for severe. (ASX: Astaxanthin, I: Ischemia, I/R: Ischemia and Reperfusion)

## Discussion

Ovarian torsion, defined as the interruption of the blood supply to the ovary, ranks fifth among all gynecological cases in women (20). It often develops during adolescence due to shortening or lengthening of the ovarian ligaments or due to ovarian tumors. Diagnosis is very important in treatment. Delayed diagnosis can cause infertility. In past cases, the only treatment was ovariectomy. In early diagnosis and treatment, the first option is to restore blood flow to the ovaries (21). Although blood flow is restored, ischemic ovarian damage and subsequent reperfusion ovarian damage are inevitable**. **Adequate oxygen cannot be transported to the tissues during ischemia and hypoxia in the ovary. Hypoxia leads to release of a number of chemicals that lead to disruption of cellular functions (22). Production of high-energy adenosine triphosphate (ATP) slows down or stops due to oxidative phosphorylation when the cell is deprived of oxygen. In this case, the main energy source of the cell is provided by anaerobic phosphorylation (22). ATP breakdown as a result of ischemia leads to accumulation of purine metabolites such as xanthine and hypoxanthine in the tissue and conversion of xanthine dehydrogenase (XDH) to xanthinoxidase (XO) (22). In normal cases, hypoxanthine is metabolized to uric acid and in this reaction the final electron acceptor is NAD^+^. However, the conversion of hypoxanthine to uric acid occurs by XO since XDH converts to XO due to hypoxia, and molecular oxygen is used as the electron acceptor in this reaction (23, 24). However, since there is no oxygen in the tissue, continuous accumulation of XO occurs in the cell. XO deposition forms the source of free radicals during reperfusion. The hypoxic is eliminated with re-flow of blood to the ovaries and toxic metabolites are removed from the tissue; however, oxygen and other metabolites that come into the tissue during reperfusion paradoxically cause tissue damage (25). Reperfusion provides oxygen to the tissue again and hypoxanthine turns into xanthine and uric acid with the enzyme XO. In the presence of oxygen, toxic compounds such as superoxide (O_2_) and hydrogen peroxide (H_2_O_2_) are formed in the tissue. All these processes develop oxidative damage in tissues. 

At this point, preventive treatment against ovarian oxidative damage is required. Natural anti-oxidant compounds have an important place in the elimination of oxidative damage. Protection of natural anti-oxidant compounds has been tested in many experimental ischemia and reperfusion models. Although studies have shown that oxidative organ damage is reduced, the incomplete removal of the damage indicates that more effective compounds or treatments are needed.

The most important feature of ASX, which is responsible for the red shell color in shellfish, is that it is an anti-oxidant superior to β-carotene and even α-tocopherol (26). Due to its outstanding anti-oxidant activity, ASX has an extraordinary potential to protect the organism against ailments such as oxidative cell damage, different types of cancer, and some diseases of the immunological system (27). Due to this feature, its effectiveness has been shown in the literature in important diseases such as aging (28), neurological diseases (29), cancer (30), and alcoholic liver damage (31). However, its efficacy against ovarian ischemia and reperfusion injury has not yet been demonstrated. For this purpose, in this study, the effect of ASX on experimental ischemia and reperfusion injury was investigated biochemically, histopathologically, and immunohistochemically.

One of the main indicators of oxidative stress in the tissue is the elevation of malondialdehyde (MDA) levels, which is the end product of lipid peroxidation. As a matter of fact, it was observed that MDA levels increased significantly in biochemical parameters of ischemia and I/R groups in our study. These results support the study named “Effect of pomegranate seed oil on ovarian ischemia/reperfusion injury in rats” (14). In I+ASX50, I+ASX100, I/R+ASX50, and I/R+ASX100 treatment groups significantly decreased MDA levels were found depending on the dose. These results are similar to the findings of the study in which Qiu *et al*. investigated the protective effect of ASX on renal ischemia-reperfusion injury in mice (11).

As it is understood, ischemia and reperfusion of the ovarian tissue is a process that needs to be managed urgently and precisely. In this process, using anti-oxidant agents before detorsion of the ovary is vital to prevent reperfusion injury. At this point, the importance of anti-oxidant systems becomes apparent. There are many protective mechanisms in biological systems to prevent the formation of free radicals and to prevent damage caused by them (32). However, the mechanisms are disturbed in acute cell damage situations that create high oxidants such as ischemia. At this stage, intrinsic anti-oxidant systems cannot be sufficient. This reveals the importance of anti-oxidant supplements (32). ASX has been proven to support intracellular anti-oxidant defense mechanisms and inactivate ROS in many studies in the literature (33).

SOD is known as a strong anti-oxidant in the cells. On the other hand, reduction of superoxide dismutase (SOD) activity, one of the intracellular anti-oxidant defense system enzymes, is also used as a reference to show oxidative stress in studies (34). As a matter of fact, it was observed that SOD levels decreased significantly in this study in I and I/R groups. On the other hand, in I+ASX50, I+ASX100, I/R+ASX50, and I/R+ASX100 treatment groups it was found that ASX significantly increased SOD levels depending on the dose. It is similar to the SOD findings of the study by Bozkurt *et al*. in which they showed the effect of selenium on ischemia/reperfusion injury in rats, and the SOD findings of the study by Islam and colleagues in which they investigated the effect of ASX in carbon tetrachloride-treated rats (35, 36).

The result of oxidative damage in tissues is histopathological tissue damage. Edema areas, hemorrhage, leukocyte infiltration, and vascular congestion findings are usually histopathologically investigated in tissue damage of ovarian ischemia-reperfusion (37-39). The areas of observed edema and hemorrhage in I and I/R groups in our study are consistent with the literature. In the treatment groups, especially in the I/R+ASX100 group, visible minimal hemorrhage areas were observed. In addition, it was noteworthy that inflammatory cells and necrotic cells were not observed in I/R+ASX50 and I/R+ASX100 groups. Similar findings were examined in the study by Ozlem *et al*. in which they investigated the effect of *Vaccinium myrtillus* in ischemia-reperfusion injury and in the study by Geyikoglu *et al*. in which they investigated the effects of propolis and boric acid on ischemia and reperfusion injury (38, 40). 

Oxidative stress can cause inflammation. Released chemokine substances during cellular destruction in the tissue called inflammatory cells. Inflammatory cells in damaged areas secrete cytokines called interleukins such as IL-1 β and IL-6 and more inflammatory cells come to the damaged area (41). In our immunohistochemical staining findings, IL-1 β and IL-6 increased the immunoreactivity response in I and I/R groups. Immune reactivity was decreased in the treatment groups. This immune reactivity is dose-dependent. This efficacy of ASX has been demonstrated in studies in the literature showing that it suppresses the production of IL-6 and IL-1 β (42, 43).

Oxidative stress and inflammation lead to cell apoptosis by cell death cascades such as Bcl-2, Caspase 9, and Caspase 3. Especially in the process until cell death, the caspase 3 (intrinsic and extrinsic pathway of apoptosis) step acts as a key molecule for apoptosis (44). From this aspect, showing the presence of the caspase 3 pathway can provide information about the severity of oxidative stress. In our Caspase 3 immunohistochemical staining findings, there was a significant immune-positivity in I and I/R groups. Indeed, in a study by Gungor *et al*., showing the effect of hesperidin on ovarian ischemia-reperfusion, Caspase 3 reactivity was increased in I and I/R groups (45). This supports our results. In treatment groups, especially in I/R+ASX50 and I/R+ASX100 groups, ASX has been shown to significantly reduce caspase 3 expression. Similar to our findings, a ischemia-reperfusion study showed that ASX reduced Caspase 3 levels (13). On the other hand, the degree of immune positivity seen in the I/R group was significantly higher than that of the ischemia group. In addition, mild positivity seen in the healthy group was found to be compatible with the literature. Especially, immune negativity in the I/R+ASX100 group indicates the effectiveness of ASX.

## Conclusion

The significant correlation observed in MDA and SOD levels in I, I/R, and treatment groups in our study were histopathologically confirmed in tissue damage. Inflammatory cell findings, especially seen in the I/R group, reflect the accuracy of immunohistochemical IL-1β and IL-6 immunopositivity results. In addition, all these findings appear to be compatible with Caspas3 levels in intergroup evaluation and reflect the concordance between our findings. All our results have been supported by the literature. In this study, we analyzed the intracellular potential protective effect of ASX on the ovary of rats by biochemical, immunohistochemical, and histopathological analysis. As a result, ASX has a strong protective role against oxidative damage in the treatment of ovarian ischemia-reperfusion injury.

## Authors’ Contributions

ET Conceived the study; ET, JS, and TBT Provided the histopathological findings; ET, OOA, and EE Provided the immunohistochemical findings; MAG Performed statistical analysis; and RAU Performed biochemical analysis.

## Conflicts of Interest

The authors declare that no conflict of interest exists.
